# Lumbosacral anatomy is unique in pediatric spondylolysis

**DOI:** 10.1007/s43390-025-01084-1

**Published:** 2025-04-03

**Authors:** Ryan A. Finkel, Nakul Narendran, Daniel Farivar, Paal Nilssen, Melodie F. Metzger, David L. Skaggs, Kenneth D. Illingworth

**Affiliations:** Cedars-Sinai Medical Center, Department of Spine Surgery, 8700 Beverly Blvd, Los Angeles, CA 90048 USA

**Keywords:** Spondylolysis, Spondylolisthesis, Pediatrics, Deformity, Low back pain

## Abstract

**Purpose:**

To determine whether patients with L5 spondylolysis have different lumbosacral anatomy compared to patients without L5 spondylolysis.

**Methods:**

Computed tomography (CT) scans of pediatric patients with isolated L5 spondylolysis were identified and matched 1:4 (age, sex, BMI) to patients without spondylolysis. Sagittal parameters assessed included sacral slope angle, sacral table angle, L4-S1 and L5-S1 Cobb angles, the horizontal angle of the L5 pars interarticularis, the distances between the L4 inferior articular process (IAP) and the S1 superior articular process (SAP) and their respective individual distances to the L5 pars. Coronal parameters assessed included the percent subluxation of L4 IAP below the facet joint.

**Results:**

1084 CT scans were reviewed. 32 patients with isolated L5 spondylolysis were identified and matched to 122 patients without spondylolysis. The horizontal angle of the L5 pars was greater in spondylolysis patients (142.5 ± 10.2 vs. 119.9 ± 5.9, *p* < 0.05). There was less distance (mm) between L4 IAP and S1 SAP (11.3 ± 3.9 vs. 14.7 ± 2.9, *p* < 0.05) and less distance (mm) from both L4 IAP (2.6 ± 1.7 vs. 5.4 ± 2.2, *p* < 0.05) and S1 SAP (0.7 ± 0.4 vs. 1.5 ± 0.7, *p* < 0.05), respectively, to the L5 pars in the spondylolysis group. Pearson’s analyses revealed that a larger horizontal angle of the L5 pars was strongly associated with spondylolysis (0.59).

**Conclusion:**

Pediatric patients with L5 spondylolysis have a significantly more horizontal L5 pars that is closer to both the L4 IAP and S1 SAP.

## Introduction

Spondylolysis is a fracture of the pars interarticularis that [1] is commonly encountered in the pediatric population [2]. It is reportedly associated with increased physical activity, with repetitive microtrauma and hyperextension cited as likely etiologies [3, 4]. Pediatric spondylolysis is one of the most common sources of low back pain in children, with a prevalence of about 6% by age six. However, the risk increases up to 47% in young athletes who participate in sports with frequent spine extension, such as gymnasts, football players and weightlifters [5, 6]. While most cases of spondylolysis are asymptomatic [7], some may progress into spondylolisthesis [8–10] and can lead to nerve root compression and radiculopathy, especially during periods of rapid bone growth [11]. Spondylolysis occurs most frequently at the L5 vertebrae, likely due to its caudal location and the biomechanical stress endured at the lumbosacral junction. The hypothesis that repetitive lumbar hyperextension creates microtrauma leading to the development of spondylolysis is supported by studies demonstrating the absence of spondylolysis at birth and absence in patients who have never ambulated [12, 13].

Diagnosis is typically made during clinical examination and confirmed via imaging modalities such as X-Ray, computed tomography (CT), single-photon emission computed tomography (SPECT) and magnetic resonance imaging (MRI) [2]. Treatment can be patient-specific but generally starts with a trial of non-operative measures including physical therapy, bracing and nonsteroidal anti-inflammatory medications [5, 12]. Surgery is generally reserved for failed nonoperative management in patients with high exertional demand [14, 15].

There is currently limited information to determine whether inherent anatomical or geometric risk factors contribute to the development of spondylolysis among pediatric patients. Therefore, the purpose of this retrospective observational study was to evaluate CT scans from pediatric patients with and without L5 spondylolysis to determine if there are any measurable differences in lumbosacral anatomy between these two patient populations. Specifically, we hypothesized that several lumbosacral morphological measurements will be significantly correlated with the risk of developing pediatric spondylolysis.

## Materials and methods

Institutional Review Board (IRB) approval was obtained prior to initiating the study. A retrospective review of all pediatric patients (< 18 years of age) with available CT scans of the lumbosacral junction was conducted between 2005 and 2022 at a single-site quaternary care center. All patients included had CT scans of the spine or abdomen and pelvis. Patients with a prior history of spinal surgery, spondylolisthesis, concurrent fractures or deformities at other levels or anatomical regions of L5 (pedicle, vertebral body, etc.) were excluded. Patients with greater than 10 degrees of scoliosis in any direction or transitional anatomy at the lumbosacral junction were also excluded. A total of 1,084 pediatric CT scans met the inclusion criteria. Two of the listed authors individually assessed each CT scan of the lumbosacral junction on axial, sagittal, and coronal views. All patients with isolated spondylolysis at L5, unilateral or bilateral, were identified. Any patient deemed to have a possible, but unclear, spondylolysis at L5 was sent to all authors for review and a decision on inclusion after final analysis was made by the senior author, a board-certified fellowship-trained pediatric spine surgeon. Demographic information was obtained including age at time of CT scan, sex, height, weight, and BMI. Pediatric patients without evidence of spondylolysis or deformity at any level were also identified and included as controls. Patients confirmed to have isolated spondylolysis at L5 were matched 1:4 to control patients by age at the time of CT scan, sex, height (cm), and weight (kg). This was done using propensity score matching of the nearest neighbor in our raw data set.

**Fig. 1:**
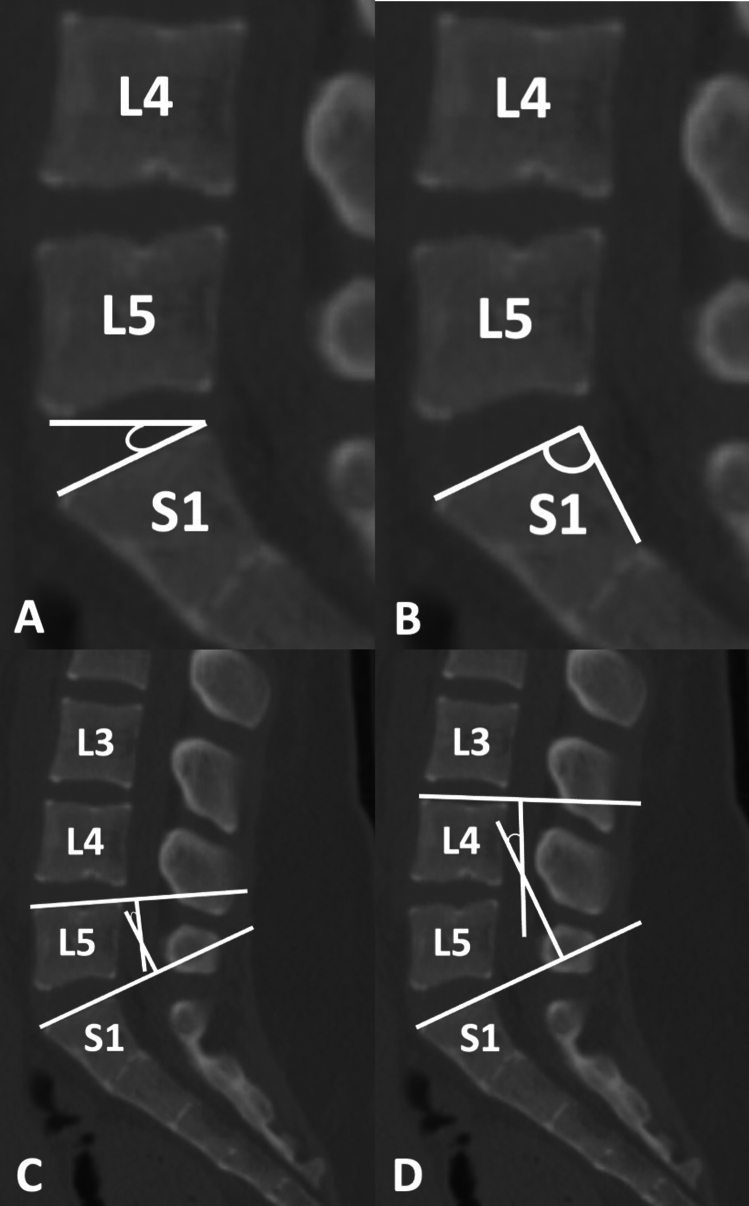
Images demonstrating sacral slope (**A**), sacral table angle (**B**), L5-S1 Cobb angle (**C**), and L4-S1 Cobb angle (**D**) measurements

**Fig. 2:**
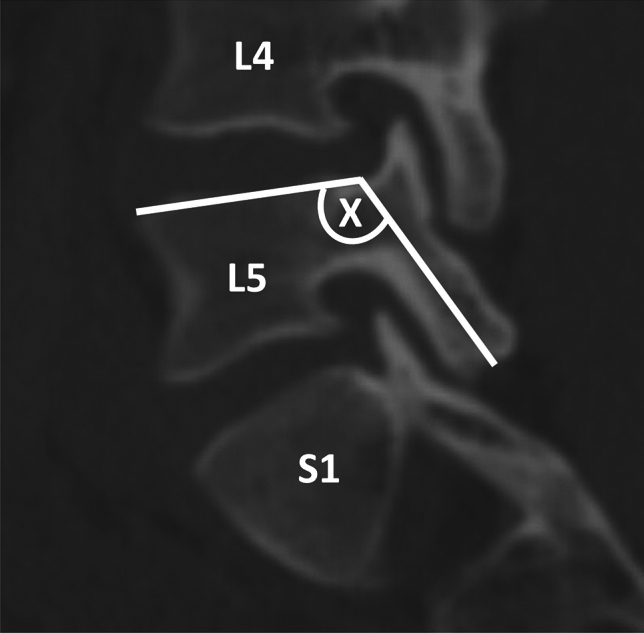
The horizontal angles of the L5 pars interarticularis (**A**) and L4 pars interarticularis (**B**)

**Fig. 3:**
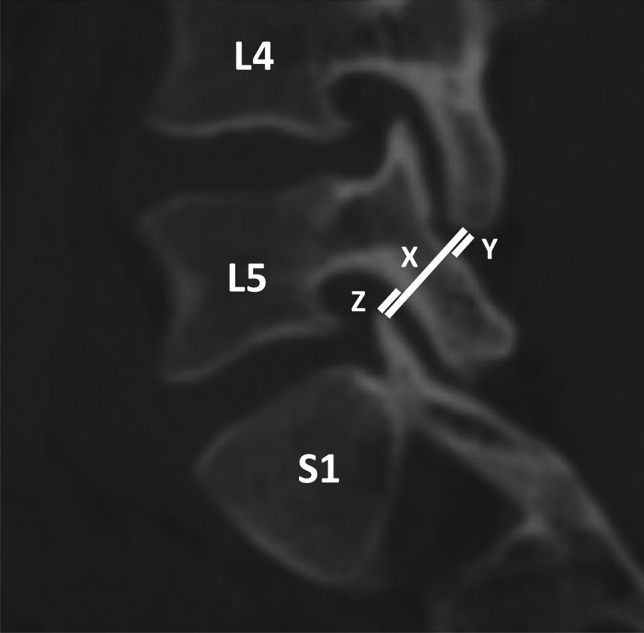
The distance between the L4 inferior articular process (IAP) and the S1 superior articular process (SAP) (X), the distance between the L4 IAP and the L5 pars interarticularis (Y), and the distance between the S1 SAP and the L5 pars interarticularis (Z)

**Fig. 4:**
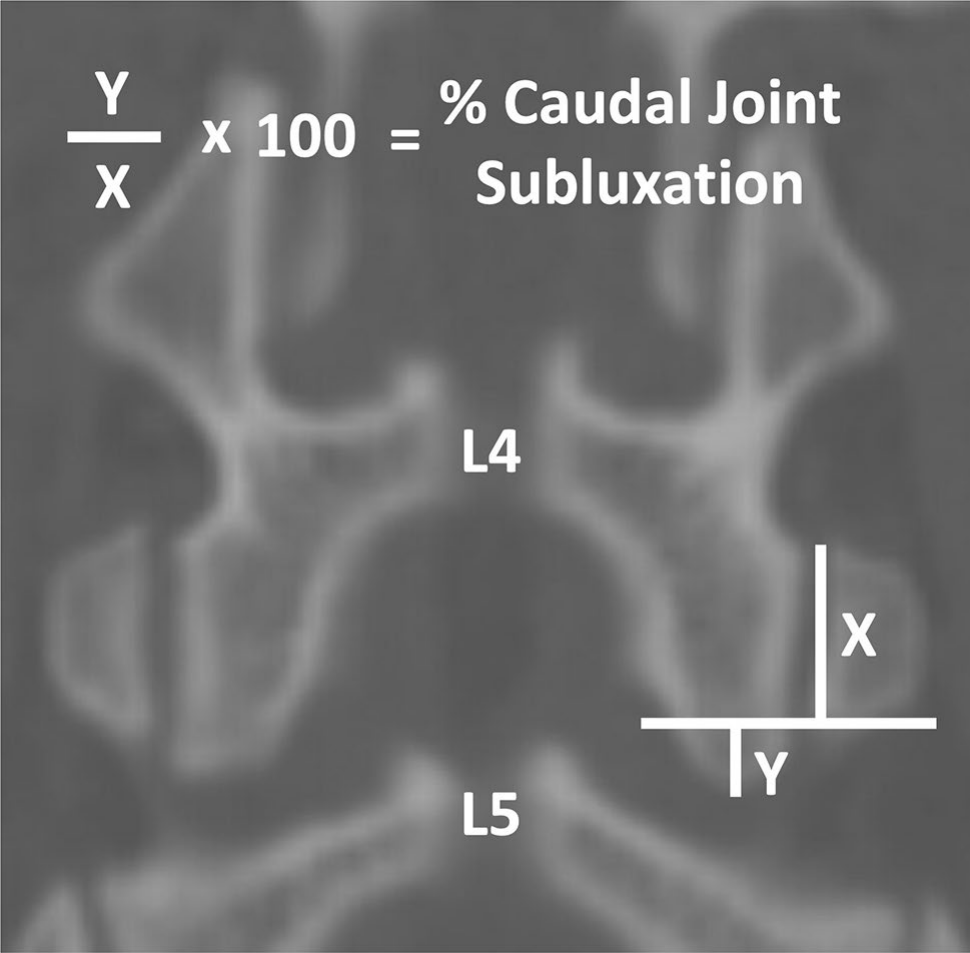
The percent subluxation of the L4 IAP below the facet joint

Lumbar spinal parameters were measured in patients from both groups (with and without isolated spondylolysis at L5) using the WebPax Picture Archive and Communications System (PACS). A total of 10 parameters were included based on midsagittal, sagittal, and coronal plane CT images. The midsagittal view of each patient’s lumbar spine was used to measure and record the following parameters: A) sacral slope angle, B) sacral table angle, C) L5-S1 Cobb angle, D) L4-S1 Cobb angle (Fig. 1). Either the left or right sagittal scans, whichever slice best bisected the pars interarticularis, was used to measure the following: (1) the angle formed by the superior endplate of L4 and a line bisecting the L4 pars interarticularis (horizontal angle of the L4 pars interarticularis), (2) the angle formed by the superior endplate of L5 and a line bisecting the L5 pars interarticularis (horizontal angle of the L5 pars interarticularis) (Fig. 2), (3) the distance between the L4 inferior articular process (IAP) and the S1 superior articular process (SAP), (4) the distance between the L4 IAP and the L5 pars, and (5) the distance between the S1 SAP and the L5 pars (Fig. 3). Finally, the coronal view was used to assess the percent subluxation of the L4 IAP below the L5 facet joint (Fig. 4).

Statistical analysis was performed using STATA software (version 14.0.371; StataCorp, LLC). For each group of patients, the mean and standard deviation of each of the 10 measured parameters was calculated. Parameters were compared using two-tailed t-tests and Pearson correlation analysis with significance defined by a *p*-value less than 0.05.

## Results

**Fig. 5:**
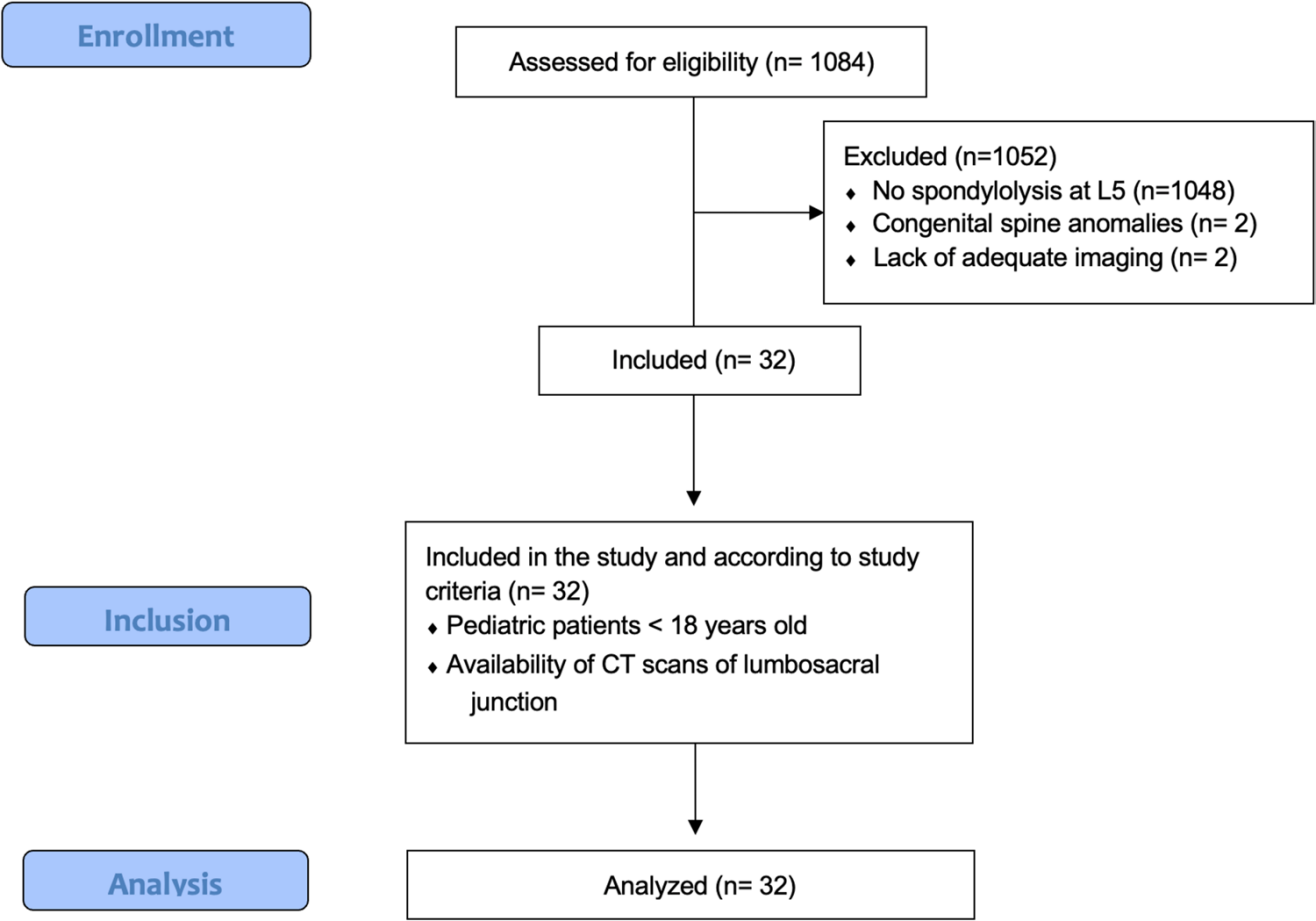
STROBE flow chart. STROBE, Strengthening the Reporting of Observational Studies in Epidemiology

36 out of 1084 (3.3%) pediatric patients with CT scans had isolated unilateral or bilateral spondylolysis at L5 **(**Fig. 5). 2 patients were subsequently excluded for congenital spine anomalies (1 patient had spina bifida occulta, 1 patient had 6 lumbar vertebra) and 2 patients were excluded for availability of only L5 CT imaging which did not allow for measurements to be made accurately, leaving 32 out of 1084 (2.9%) who fit inclusion criteria and were included in the current study’s analysis. These patients were matched to 122 control patients that met the inclusion criteria. 15 of 32 (46.9%) spondylolysis patients were female, and 52 of 122 (42.6%) control patients were female. The mean age, proportion of females, height, and weight of patients were not statistically different between the two groups (Table 1).

**Table 1:** Demographic data

	Spondylolysis	Stdev	Controls	Stdev	*P* value
Age (years)	15	2.31	14.99	1.96	0.98
Height (cm)	164.69	14.61	165.41	11.33	0.77
Weight (kg)	62.64	18.02	63.97	17.0	0.70

The horizontal angles of the L5 pars interarticularis and L4 pars interarticularis were significantly greater in patients with spondylolysis (Table 2). Pearson’s analyses revealed that a larger horizontal angle of the L5 pars interarticularis was strongly associated with isolated L5 spondylolysis (0.6). In the 10 cases of unilateral spondylolysis, the horizontal angles of the L5 pars interarticularis (145.5° ± 10.9° vs. 136.5° ± 15.9°; *p* = 0.157) and L4 pars interarticularis (109.7° ± 4.1° vs. 110.5° ± 5.6°; *p* = 0.719) on the affected sides were not significantly different than the unaffected sides (i.e. right versus left). The overall L4 IAP to S1 SAP distance, as well as the sub-measurements of L4 IAP to L5 pars and S1 SAP to L5 pars, were all significantly smaller in patients with spondylolysis. Patients with spondylolysis also had a significantly greater degree of subluxation of the L4 IAP beneath the facet joint on coronal views. There were no differences in sacral slope angle, sacral table angle, L4-S1 Cobb angle, or L5-S1 Cobb angle between patients with and without a spondylolysis.

**Table 2:** Raw measurement data

	Spondylolysis	Stdev	Controls	Stdev	*P* value
Sacral slope (deg)	37.5	9.76	36.2	7.98	0.42
Sacral table (deg)	102.59	8.33	99.4	10.1	0.10
L4-S1 cobb (deg)	36.19	8.54	35.53	6.49	0.64
L5-S1 cobb (deg)	23.06	6.24	22.98	5.22	0.94
L5 pars angle (deg)	142.52	10.18	119.93	5.85	0.01
L4 IAP to L5 pars (mm)	2.59	1.66	5.36	2.21	0.01
S1 SAP to L5 pars (mm)	0.71	0.40	1.48	0.66	0.01
L4 IAP to S1 SAP (mm)	11.33	3.89	14.71	2.92	0.01
L4 pars angle (deg)	111.3	9.59	108.12	5.92	0.02
% Subluxation	29.03	20.1	13.18	11.38	0.01

## Discussion

The most important finding of this study was that several morphological measurements of the lumbosacral junction predispose pediatric patients to the development of spondylolysis. Specifically, patients with spondylolysis had significantly greater caudal L4/L5 facet joint subluxation and an increased horizontal angle of the L5 pars interarticularis, two measurements that have not previously been described or evaluated in the medical literature.

Previous studies that have evaluated the potential etiologies of spondylolysis have largely focused on spinopelvic parameters. In a retrospective study that included 13 different centers, Labelle et al. found that PI was significantly higher in teenage, adolescent and young adult patients with L5-S1 spondylolisthesis (*n* = 214) compared to normal subjects (*n* = 160) [4], which was confirmed in subsequent studies [16–19]. In addition, others have reported that PI is positively correlated with spondylolisthesis severity [4, 20–22]. Increased sacral slope and lumbar lordosis have also been identified in patients with spondylolysis compared to normal subjects [4, 16–18]. Since PI is a fixed value, Stagnara et al. suggested that increased PI is directly related to an increased SS and that any increases in LL are compensatory to maintain a proper center of gravity [23]. A later study evaluating 198 young adult patients did not find an associated increase in LL with PI and SS but hypothesized their patients were too young to develop the compensatory mechanism [19]. Multiple studies have also determined that patients with spondylolysis have a significantly lower sacral table angle compared to control patients [19, 24, 25]. Despite the aforementioned findings relating spondylolysis to spinopelvic parameters, there is little known about the role of L5 vertebral anatomical variations in patients with spondylolysis.

**Fig. 6:**
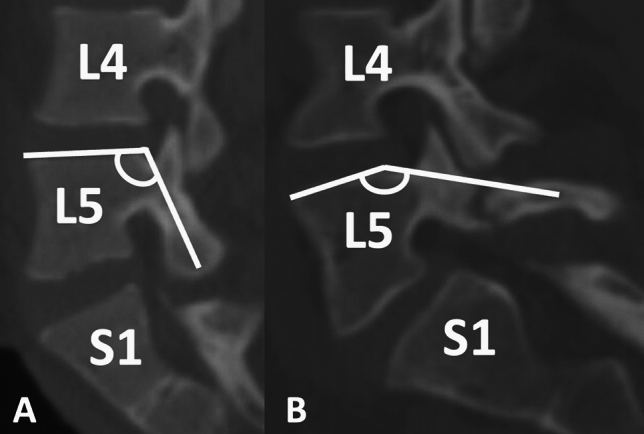
Comparison of a patient without spondylolysis and a **A** shallow horizontal angle of the L5 pars interarticularis to a **B** patient with spondylolysis and a flat horizontal angle of the L5 pars interarticularis

The present study determined that pediatric patients with L5 spondylolysis are more likely to have a horizontal L5 pars situated closer to both the L4 IAP and S1 SAP (Fig. 6). The present study is the first investigation to evaluate inherent geometric lumbar and lumbosacral variations in patients with spondylolysis compared to matched controls. It is well described that PI does not change with seated versus standing positions, but there is literature to suggest that other lumbar and sacral anatomic parameters such as LL and SS change depending on the position of the patient [26]. The present study provides another set of parameters that are inherent to the patients’ native anatomy regardless of their body position. The horizontal angle of the L5 pars is defined solely by the superior endplate of L5 and the angle subtended by the midpoint of the pars of the same vertebral segment, which is a fixed parameter and does not change based on positioning. We postulate that an increase in the horizontal angle of the L5 pars predisposes patients to a more frequent abutment to the cranial and caudal facet joints during weight-bearing, rotational and hyperextension activity. It is also likely that patients with increasingly horizontal L5 pars and decreased distance between the cranial and caudal facet joints have increased forces across the L5 pars during weight-bearing activity and lumbar extension due to a shortening of the facet distance cranially and caudally. Furthermore, anatomical differences may also contribute to the frequency of spondylolysis at L5 compared to more cephalad vertebrae. In the current study, the horizontal angle of the L4 pars interarticularis in patients with spondylolysis was on average 111.3 ± 9.5°, which was substantially less than the value of its L5 counterpart of 142.5 ± 10.1°. Many factors unique to L5 and the lumbosacral junction may predispose L5 to a higher incidence of spondylolysis, and having a substantially larger horizontal angle at the pars interarticularis likely plays an important role.

**Fig. 7:**
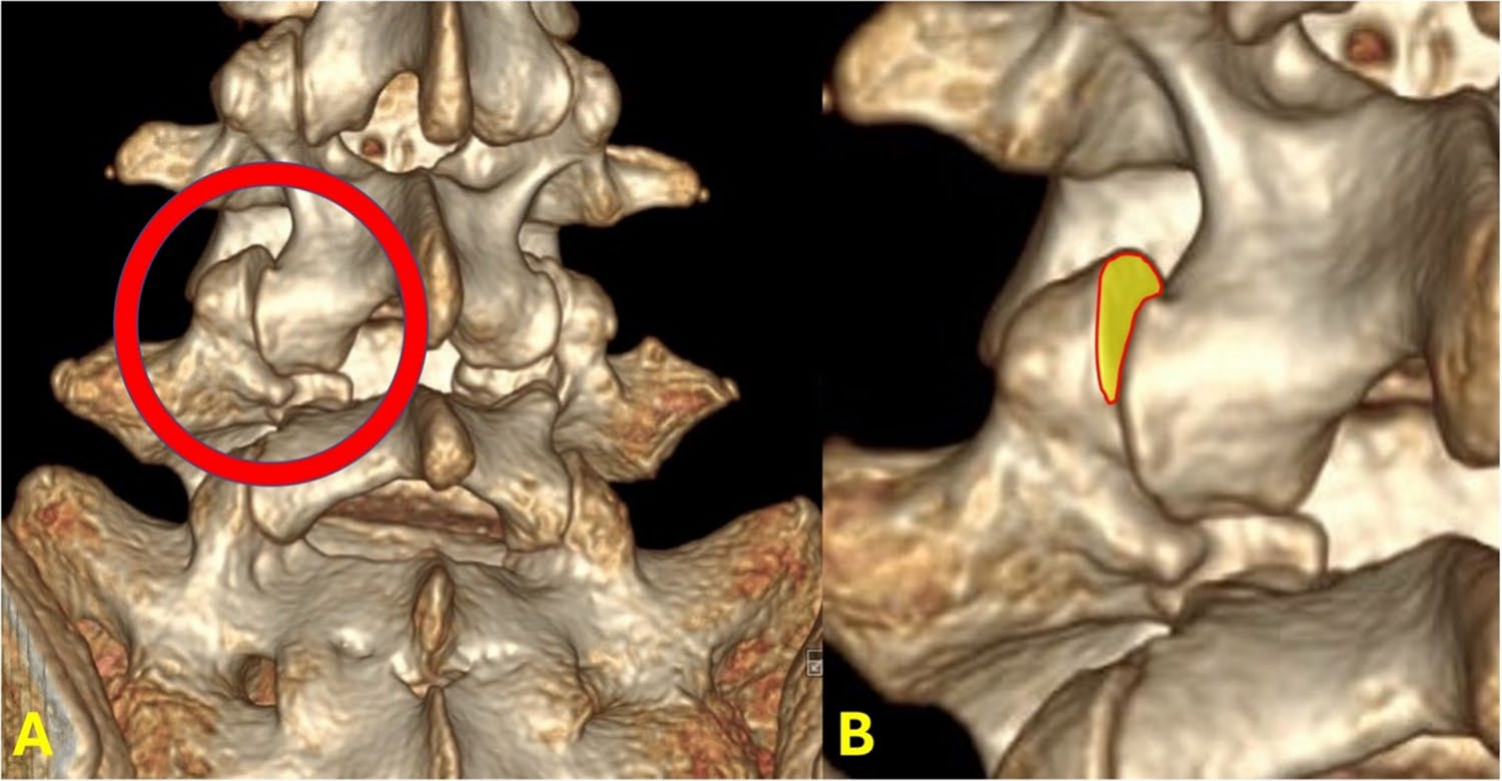
A three-dimensional reconstruction of computed tomography (CT) images, illustrating the L4 inferior articular process (IAP) subluxating below the L4/L5 facet joint

In addition to the horizontal angle of the L5 pars interarticularis, we quantified L4/L5 joint subluxation (Fig. 7). The etiology of pediatric spondylolysis is multifactorial, with a combination of genetic, morphological and activity-related factors which play a role in its development. Our work suggests L4/L5 joint pathology is a possible contributor to the development of spondylolysis as well, but it does not comment on the causal relationship of these variables. Lumbar facet tropism has been defined as unequal rotation in the axial plane which subsequently can lead to asymmetry in the appearance of the joint. Some studies have attributed a more sagittal-oriented facet joint to the likelihood of having spondylolisthesis. Our study did not particularly evaluate the orientation of the lumbar facet joints but it is important to note that these correlations have been made in previously published literature [27, 28]. The current authors believe that the vertebra in children with a higher pelvic incidence and compensatory lumbar lordosis may undergo morphological changes over time from altered stress patterns that result in a more horizontal pars angle, and ultimately predispose to spondylolysis. However, future studies will be needed to examine the merit of such a pathogenesis.

This retrospective study had several limitations. Our institution has a standardized trauma CT protocol that is different from the thin cuts obtained during dedicated lumbar spine CT scans for spinal pathology evaluation. As a result, the distance between cuts was not uniform throughout the data set. Some CT scans employed a musculoskeletal (MSK) protocol (1 mm slices), while others employed a trauma protocol with wider cuts (2 + mm slices). Although the present study intended to establish static and inherent geometric differences between patients that would not change with positioning, there was no uniform weightbearing assessment of spinopelvic and lumbosacral alignment. This would not affect the measurements of the L5 pars angle but may influence the distance between the cranial and caudal facet joints with respect to the L5 pars. Furthermore, it was assumed that the distance between the L4 IAP and S1 SAP was negligible based on the CT scan protocol used. Additionally, instability at the site of spondylolysis may contribute to the horizontal angles at the L4 and L5 pars interarticularis, rather than inherent morphology of the particular patient. However, in our study, the horizontal angles of the pars interarticularis on the spondylolysis sides and unaffected sides were not found to be statistically different. It must also be mentioned that standing CT scans were not available at the institution where this study was performed, and so supine CT scans may have potentially masked subtle cases of spondylolisthesis that weight-bearing stress could have demonstrated. Lastly, maturation and ossification of the vertebral endplates varies by age, which may have created variations in anatomical measurements within our population [29].There are three ossification centers in the lumbar vertebral bodies. In general, the fusion of these primary ossification centers occurs by 6 years old, but the cranial and caudal margins of the vertebral bodies can fuse anywhere from 14 to 24 years of age [29]. Thus, our study, with a range of patients aged 10–18, included patients with slight variations in endplate anatomy. In these subjects, we were able to acquire appropriate measurements by carefully adjusting the contrast as needed to adequately visualize the cranial ring apophysis at L5.

In conclusion, the current study determined that pediatric patients with L5 spondylolysis had caudal subluxation of the L4/L5 facet joint and had a significantly more horizontal L5 pars situated closer to both the L4 IAP and S1 SAP. Future studies focused on the clinical implications of these geometric and anatomic differences in at-risk patients are needed.

## Data Availability

The data supporting the findings of this study are available from the corresponding author upon reasonable request.
